# Adjuvant Nivolumab Therapy for Upper Tract Urothelial Carcinoma: A Single-Institution Study

**DOI:** 10.7759/cureus.89897

**Published:** 2025-08-12

**Authors:** Akinori Minato, Katsuyoshi Higashijima, Yui Mizushima, Yoshihiro Sugita, Kazumasa Jojima, Rieko Kimuro, Yujiro Nagata, Ikko Tomisaki, Eiji Kashiwagi, Naohiro Fujimoto

**Affiliations:** 1 Department of Urology, University of Occupational and Environmental Health, Kitakyushu, JPN; 2 Department of Urology, Kurate Hospital, Kurate, JPN

**Keywords:** adjuvant chemotherapy, adjuvant immunotherapy, adjuvant nivolumab, immune checkpoint inhibitor, muscle-invasive urothelial carcinoma, neoadjuvant chemotherapy, radical nephroureterectomy, upper tract urothelial carcinoma

## Abstract

Background: While immune checkpoint inhibitors have transformed adjuvant therapy for urothelial carcinoma, data focused on upper tract urothelial carcinoma (UTUC) remain scarce. In the CheckMate 274 trial, the UTUC subgroup did not demonstrate a definitive survival benefit. This study aimed to evaluate the clinical outcomes of adjuvant nivolumab in patients with high-risk UTUC.

Patients and methods: Of the 41 patients who underwent radical nephroureterectomy for UTUC at our institution between April 2022 and April 2024, 11 received adjuvant nivolumab, thereby retrospectively analyzed. Their treatment adherence and immune-related adverse events (irAEs) and survival outcomes were assessed.

Results: Of the 11 patients, eight patients (72.7%) received neoadjuvant chemotherapy. Seven (63.6%) completed the 12-month nivolumab regimen. Four discontinued the treatment because of irAEs (n = 2) and disease recurrence (n = 2). None experienced treatment-related death. The one- and two-year metastasis-free survival rates were 81.8% and 70.1%, respectively, with a median of 30 months. The two-year disease-free and overall survival rates were 40.9% and 79.5%, respectively.

Conclusion: This is one of the first real-world experiences focused exclusively on patients receiving adjuvant nivolumab for UTUC. The observed delay in distant progression may reflect the systemic therapeutic activity of immune checkpoint blockade in this setting.

## Introduction

Upper tract urothelial carcinoma (UTUC) accounts for only 5%-10% of all urothelial carcinomas, characterized by distinct embryological origin, molecular features, and clinical behavior compared with its bladder counterpart [1-3]. Although radical nephroureterectomy (RNU) remains the gold-standard treatment for localized UTUC [4], cisplatin-based perioperative chemotherapy is often limited to the disease’s rarity combined with age-related comorbidities and impaired renal function [5-7]. Despite advances in metastatic treatment, such as enfortumab vedotin plus pembrolizumab, gemcitabine plus cisplatin with nivolumab, and erdafitinib, strategies that can prevent or delay recurrence following curative-intent surgery remain urgently needed [8-10]. However, recurrence, both local and distant, is frequently observed, especially in patients with advanced pathological features such as pT3 or nodal involvement [4,11]. Thus, systemic adjuvant therapy has been explored to reduce recurrence risk and improve long-term outcomes [12]. The POUT trial established adjuvant platinum-based chemotherapy as a standard treatment option for high-risk UTUC, demonstrating improved disease-free survival (DFS) [13].

More recently, the CheckMate 274 trial evaluated adjuvant immune checkpoint blockade using nivolumab in patients with high-risk urothelial carcinoma [14]. While this drug demonstrated improved survival in all participants, its relevance to UTUC remains uncertain because patients with UTUC were few and the subgroup results were inconclusive [15]. These limitations underscore a persistent evidence gap in UTUC-specific adjuvant strategies, contrasting sharply with the rapidly expanding immunotherapy landscape in bladder cancer [16].

This study aimed to evaluate the feasibility, safety, and oncologic outcomes, including both DFS and metastasis-free survival (MFS), of adjuvant immune checkpoint blockade in real-world patients with UTUC, offering preliminary data to guide future UTUC-specific immunotherapy strategies.

## Materials and methods

Patient selection

This retrospective study included patients who underwent RNU for UTUC treatment at the University of Occupational and Environmental Health (UOEH) Hospital between April 2022 and April 2024. Among the 41 consecutive patients, those with pathological features meeting the high-risk criteria, defined as pT3-4 or pN-positive disease per the CheckMate 274 trial, were eligible for adjuvant nivolumab [14]. This study obtained approval from the UOEH’s institutional review board (approval no.: CRG25-012) and conformed to the principles of the Declaration of Helsinki.

Treatment protocol

Patients with malignancy clinically staged as cT3 or higher and/or with radiological lymph node involvement received platinum-based neoadjuvant chemotherapy (NAC), which involved three planned cycles. Those with cT2 or lower and clinically node-negative disease proceeded directly to RNU. Template-based regional lymph node dissection (LND) was routinely performed according to the approach described by Kondo et al. [17].

Data collection and outcomes

Clinical and pathological data were retrospectively extracted from the medical records. Postoperative surveillance included cross-sectional imaging, typically contrast-enhanced computed tomography of the chest, abdomen, and pelvis when renal function was adequate, and cystoscopy every three months for the first two years and every six months thereafter. Additional imaging was performed when clinically indicated. Distant metastases were confirmed radiologically based on computed tomography, and intravesical recurrences were confirmed histologically via transurethral resection or biopsy.

Furthermore, treatment adherence, immune-related adverse events (irAEs), and oncologic outcomes were assessed. irAEs were graded according to the Common Terminology Criteria for Adverse Events, version 5.0 [18], and were managed under the supervision of the institutional irAE management committee, including temporary treatment interruption, corticosteroid administration, and supportive care as appropriate. The primary endpoints included DFS, MFS, overall survival (OS), and intravesical recurrence-free survival (IVRFS). PD-L1 testing was not performed because it was not routinely available in the Japanese clinical setting during the study period.

Statistical analysis

All statistical data were analyzed using EZR version 1.55 (Easy R, Saitama Medical Center, Jichi Medical University, Saitama, Japan), a graphical user interface for R [19]. The interval from surgery to recurrence at any site or death is defined as DFS. The time to the development of distant metastasis or death indicated MFS. OS was calculated from surgery to death from any cause. IVRFS was defined as the time to histologically confirmed recurrence within the bladder or death. Additionally, survival curves were estimated using the Kaplan-Meier method. The median survival durations and 95% confidence intervals (CIs) were reported for each endpoint.

## Results

Patient characteristics

This study included 11 patients who received adjuvant nivolumab following RNU for UTUC. Table 1 lists the baseline clinicopathological characteristics.

**Table 1:** Baseline clinicopathological patient characteristics at the initiation of adjuvant nivolumab therapy ECOG PS: Eastern Cooperative Oncology Group performance status, UC: urothelial carcinoma, MVAC: methotrexate, vinblastine, doxorubicin, and cisplatin, eGFR: estimated glomerular filtration rate.

Characteristic	N = 11
Age, median (range)	72 (60–84)
Sex, n (%)	
Male	9 (81.8)
Female	2 (18.2)
ECOG PS score, n (%)	
0	11 (100)
Primary tumor site, n (%)	
Renal pelvis	8 (72.7)
Ureter	3 (27.3)
Histology, n (%)	
Pure UC	9 (81.8)
UC subtype	2 (18.2)
Clinical tumor stage, n (%)	
Ta, T1	2 (18.2)
T2	2 (18.2)
T3	7 (63.6)
Clinical nodal status, n (%)	
Positive	3 (27.3)
Previous neoadjuvant chemotherapy, n (%)	
Gemcitabine + cisplatin	1 (9.1)
Gemcitabine + carboplatin	4 (36.3)
Dose dense-MVAC	3 (27.3)
Not administered	3 (27.3)
Regional lymph node dissection, n (%)	
Yes	11 (100)
Pathological stage, n (%)	
pT3N0	7 (63.6)
pT4N0	1 (9.1)
Any pT and N positive	3 (27.3)
Median eGFR, ml/min/1.73 m^2 ^(range)	41 (21–67)
Follow-up period, median months (range)	20 (7–36)

The median age was 72 years, with two female patients included. The primary tumor was located in the renal pelvis in eight patients and the ureter in three. Eight patients (72.7%) had received prior NAC. All patients underwent regional LND at the time of surgery. The pathological stage was pT3-4N0 or any pT with nodal involvement, corresponding to a high-risk disease.

Nivolumab was administered intravenously at 240 mg/body every two weeks or 480 mg/body every four weeks, for a planned duration of 12 months. The treatment was discontinued if distant metastasis or clinically significant irAEs developed.

Treatment adherence and the clinical course

The median follow-up duration from surgery was 20 (7-36) months. Seven patients (63.6%) completed the 12-month nivolumab regimen. Four patients discontinued treatment because of irAEs and recurrence (two patients each). Importantly, all patients had completed the full 12-month observation period, enabling a definitive and uncensored assessment of treatment adherence, toxicity, and survival outcomes. Table 2 summarizes the treatment courses. Among the five patients (45.5%) who experienced intravesical recurrence, all lesions were non-muscle-invasive urothelial carcinoma. Three patients (27.3%) developed distant metastases. In cases of intravesical recurrence during the protocol period, adjuvant nivolumab therapy was continued as planned, and intravesical BCG therapy was not administered concurrently.

**Table 2:** Summary of treatment courses and clinical outcomes in 11 patients receiving adjuvant nivolumab therapy irAE: Immune-related adverse event.

Parameter	Value
Exposure period of nivolumab, median months (range)	12 (5–12)
Treatment completion, n (%)	7 (63.6)
Treatment interruption caused by irAEs, n (%)	3 (27.3)
Treatment discontinuation, n (%)	4 (36.4)
─ IrAEs	2 (18.2)
─ Disease recurrence	2 (18.2)
Corticosteroid administration for irAEs	2 (18.2)
Recurrence, n (%)	
─ Intravesical	5 (45.5)
─ Distant metastasis	3 (27.3)
Cause of death, n (%)	
─ Disease progression	2 (18.2)
─ Other	2 (18.2)

Immune-related adverse events

Five patients (45.5%) experienced irAEs, among whom three (27.3%) experienced grade ≥3 events, specifically hepatitis, skin toxicity, and amylase level elevation. Two patients required corticosteroid therapy, and no grade 4 or 5 irAEs occurred. The most common irAE was hypothyroidism, observed in two patients (18.2%), though all were grade 1-2.

Survival outcomes

**Figure 1:**
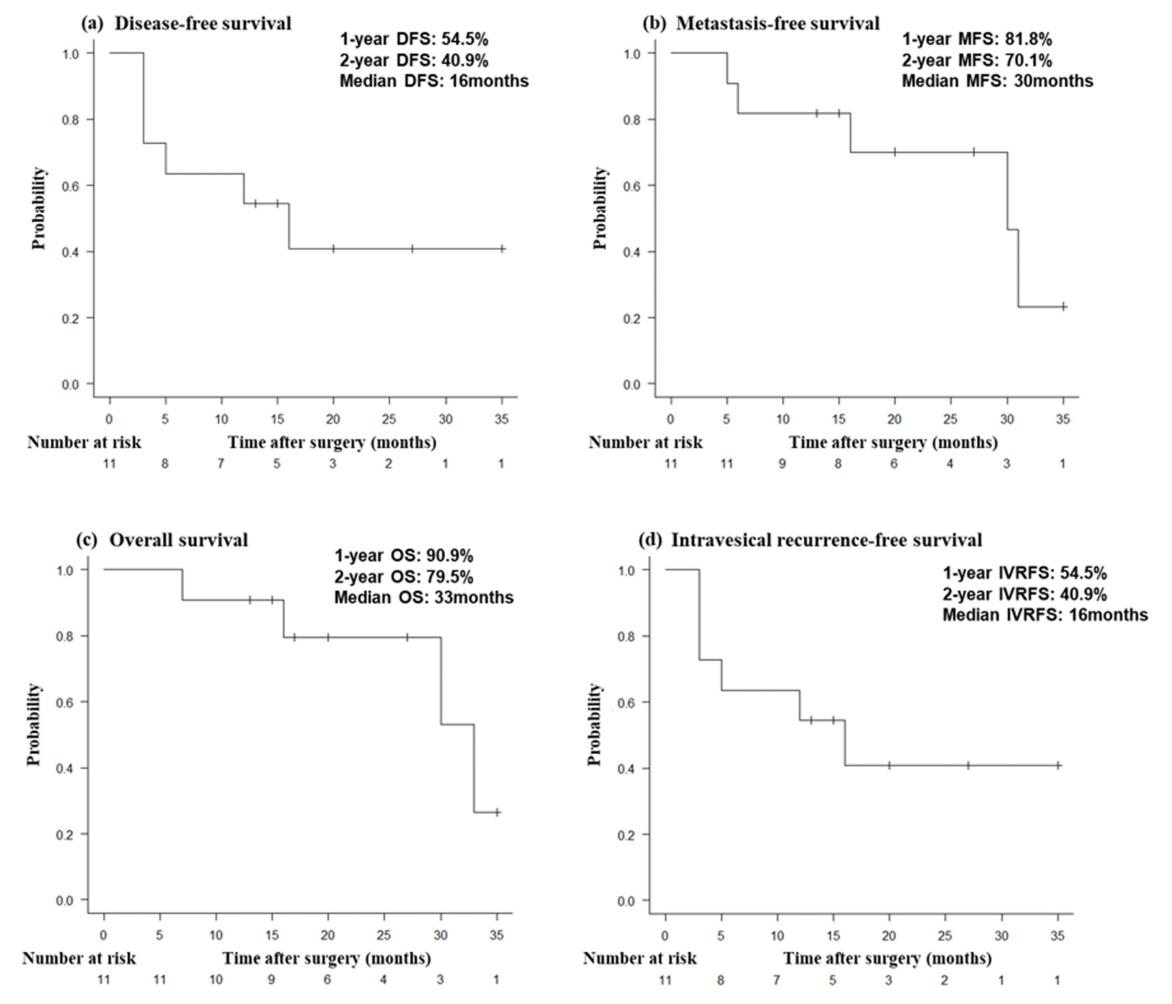
Kaplan–Meier survival curves in patients with upper tract urothelial carcinoma receiving adjuvant nivolumab. (a) Disease-free survival (DFS), (b) metastasis-free survival (MFS), (c) overall survival (OS), and (d) intravesical recurrence-free survival (IVRFS). Note: In this cohort, the DFS and IVRFS curves are identical because initial recurrence events were intravesical and occurred either before or concurrently with distant metastases. None of the patients experienced local pelvic or distant recurrence prior to bladder recurrence.

Figure 1 illustrates the survival analyses. The two-year DFS and OS rates were 40.9% and 79.5%, with median durations of 16 and 33 months (95% CI, 3.0- not estimable (NE) and 16-NE), respectively (Figures 1a, 1c). Notably, the one- and two-year MFS rates were 81.8% and 70.1%, respectively, with a median of 30 months (95% CI, 6.0-NE) (Figure 1b). Furthermore, the two-year IVRFS rate was 40.9% (95% CI, 3.0-NE) (Figure 1d).

## Discussion

This study provides one of the first real-world investigations focusing exclusively on patients with UTUC treated with adjuvant immune checkpoint blockade. Results showed that 63.6% of 11 patients with high-risk UTUC successfully completed the planned 12-month nivolumab regimen following RNU. The treatment was generally well tolerated with no severe irAEs, with corticosteroids required in only two patients. Notably, the two-year MFS rate was 70.1%, with a median duration of 30 months, indicating that the adjuvant therapy can potentially delay distant progression.

Adjuvant nivolumab significantly prolonged DFS in the overall population of the CheckMate 274 trial, reducing the mortality risk by 24%; however, the UTUC subgroup, which comprised only 21% of enrolled patients, did not show a clear survival benefit [14]. This discrepancy may be attributed to several factors, including limited statistical power, differing recurrence patterns, and inherent biological divergence from bladder cancer [1,2,16]. While upper tract recurrence is relatively uncommon after radical cystectomy in patients with bladder cancer, intravesical recurrence frequently occurs in patients with UTUC following RNU [20,21], possibly disproportionately impacting DFS without necessarily reflecting systemic failure. In the present study, such recurrences were relatively common, highlighting systemic therapy’s limitation in preventing urothelial field effects. Interestingly, in our cohort, all observed recurrences initially manifested as intravesical lesions, without any cases of local pelvic or distant recurrence preceding bladder relapse. As a result, the Kaplan-Meier curves for DFS and IVRFS showed complete overlap. However, these events, although clinically inconvenient, are generally less prognostic than distant progression [22]. Against this background, our MFS results appear favorable relative to historical outcomes, suggesting a meaningful delay in distant progression. Given that distant metastasis chiefly determines UTUC prognosis [23], this outcome highlights the systemic relevance of adjuvant immunotherapy in the micrometastatic setting [24]. Therefore, in UTUC, MFS may serve as a more suitable surrogate endpoint than DFS when assessing the efficacy of systemic therapy.

Our UTUC-only cohort offers a particularly informative real-world perspective, owing to the standardized application of perioperative strategies. In our institutional protocol, platinum-based NAC, which typically consists of three cycles, was routinely administered to patients with malignancy clinically staged as T3 or node-positive. Interestingly, NAC reportedly enhances the efficacy of subsequent immune checkpoint blockade by inducing immunogenic priming, thereby augmenting the antitumor immune responses [24].

Intraoperatively, all patients underwent template-based LND [17], ensuring consistent and accurate pathological staging, a prerequisite for optimal risk stratification and informed adjuvant decision-making. This methodological consistency enhanced the internal validity of our survival analyses. Conversely, LND was performed in only 32% of patients in the POUT trial and 73% in the CheckMate 274 trial [13,14], highlighting a source of heterogeneity that may limit external comparisons.

Unlike previous retrospective series involving patients with muscle-invasive urothelial carcinoma who had incomplete treatment follow-up or were still ongoing therapy during the analysis [25,26], our cohort exclusively comprised individuals who had either completed or discontinued the full 12-month regimen. Yasuda et al. reported the efficacy of adjuvant nivolumab in a mixed cohort of patients with bladder and UTUC in Japan, with only eight UTUC cases and a median follow-up of 11 months [26]. Although informative, the findings from that study may have limited applicability to UTUC-specific outcomes, as the study was not focused solely on upper tract disease in the selected cohort.

In CheckMate 274, treatment discontinuation caused by irAEs occurred in 17% of patients, with grade 3-4 toxicities such as hepatic dysfunction and endocrinopathies being among the most concerning [14]. Similarly, a recent real-world analysis reported a 22.2% discontinuation rate, citing interstitial pneumonitis and hepatotoxicity as major complications necessitating clinical vigilance [26]. By comparison, our discontinuation rate was 18.2%, with no Grade 4 or 5 irAEs, indicating that our safety profile is generally well tolerated. These results support the feasibility of adjuvant nivolumab administration in well-selected patients with UTUC managed under structured surveillance protocols.

In clinical practice, the benefit of adjuvant chemotherapy for muscle-invasive urothelial carcinoma remains controversial because of conflicting data across anatomical sites and patient subsets [23,27]. The POUT trial, which is the only randomized controlled study specific to UTUC, demonstrated a clear DFS advantage for platinum-based chemotherapy, particularly in pT3/4N0 disease but not in node-positive cases [15]. Moreover, impaired renal function following nephroureterectomy often precludes cisplatin-based chemotherapy, further limiting the applicability of this approach in real-world patients with UTUC [6]. Hence, optimal treatment selection criteria and sequencing strategies for adjuvant chemotherapy versus immune checkpoint blockade in UTUC need to be established. A rational, biomarker-driven approach may help personalize adjuvant therapy in this heterogeneous disease [3,4].

This study has inherent limitations, including its retrospective design, limited sample size, and single-institution setting, restricting the generalizability of the findings. Additionally, the absence of a control arm prevents definitive conclusions regarding the causal efficacy of adjuvant immune checkpoint blockade. Although follow-up duration may be sufficient to observe early recurrence events, extended observation is necessary to assess durable survival benefits. Furthermore, PD-L1 testing, which is often used to inform immunotherapy decisions in clinical trials, was not routinely available in the Japanese clinical setting during the study period. Finally, the retrospective design and the fact that only patients meeting our institutional eligibility criteria for adjuvant nivolumab were included may have introduced selection bias.

Nevertheless, our study has distinct methodological strengths. It focuses exclusively on UTUC, a rare and underrepresented subset. It also features consistent application of NAC and template-based LND. Moreover, all patients had completed or discontinued the planned 12-month nivolumab regimen during the analysis, ensuring full ascertainment of treatment adherence, irAEs, and survival outcomes without censoring-related bias.

## Conclusions

Adjuvant nivolumab therapy following RNU was feasible and well tolerated in a small real-world cohort of patients with high-risk UTUC. Our findings may offer preliminary insights into the potential role of immune checkpoint blockade in this setting, particularly in the control of micrometastatic disease. Although larger, prospective, multicenter trials are required for the definitive validation of this approach, our results offer valuable insights to inform current clinical decision-making and the design of future UTUC-specific studies.
